# Mechanistic Insights Into NFIX‐Mediated DNA Recognition and Transcriptional Regulation in Skeletal Muscle

**DOI:** 10.1002/smmd.70027

**Published:** 2026-01-29

**Authors:** Ci Zhu, Shuang Liu, Xi Chen, Chengxiao Qin, Yueyu Wang, Chunchun Xue, Lingxing Li, Wenlan Du, Xin Chen, Xiaofeng Li, Jie Shen, He Song

**Affiliations:** ^1^ State Key Laboratory of Mechanism and Quality of Chinese Medicine Institute of Chinese Medical Sciences University of Macau Macau China; ^2^ Shanghai Municipal Hospital of Traditional Chinese Medicine Shanghai University of Traditional Chinese Medicine Shanghai China; ^3^ Department of Orthopedic Surgery School of Medicine Washington University St. Louis Missouri USA

**Keywords:** DNA recognition, homeostasis, NFIX, skeletal muscle development, transcriptional regulation

## Abstract

Skeletal muscle is essential for voluntary movement and exhibits a remarkable capacity for regeneration following injury. NFIX, a member of the Nuclear Factor I (NFI) family of transcription factors, plays a critical role in both skeletal muscle development and regeneration. Despite its emerging importance, the molecular basis of NFIX‐mediated DNA recognition and transcriptional regulation in skeletal muscle remains poorly defined. Here, we demonstrate that NFIX promotes key cellular processes in skeletal muscle cells, as siRNA‐mediated knockdown of NFIX significantly reduces cell proliferation, increases apoptosis, and impairs differentiation. Transcriptomic analysis revealed that NFIX regulates a network of genes involved in muscle metabolism, stress responses, and immune inflammatory responses. Biophysical characterization showed that NFIX exists as a monomer in solution and binds palindromic DNA with a 1:1 stoichiometry. A high‐resolution crystal structure of the NFIX_DBD_ bound to palindromic DNA reveals a monomeric binding mode driven by base‐specific recognition of the TGGCA motif. Mutations that disrupt key DNA‐contacting residues abolished both DNA binding and transcriptional activation in luciferase reporter assays. Together, these findings define the molecular mechanism of NFIX‐dependent gene regulation in skeletal muscle and establish a structural framework for its function, providing new insights into the potential therapeutic targeting of NFIX in muscle diseases.

## Key Points

- An atomic view of the NFIX–DNA complex uncovers its role in skeletal muscle development and disease.
- NFIX directs muscle‐related transcriptional programs via sequence‐specific recognition of the TGGCA motif.
- Integrated biophysical and structural analyses provide the molecular basis of TGGCA major‐groove recognition by NFIX.

## Graphical abstract

An atomic view of the NFIX–DNA complex reveals recognition of the TGGCA motif and links sequence‐specific binding to the transcriptional programs governing skeletal muscle development and disease.

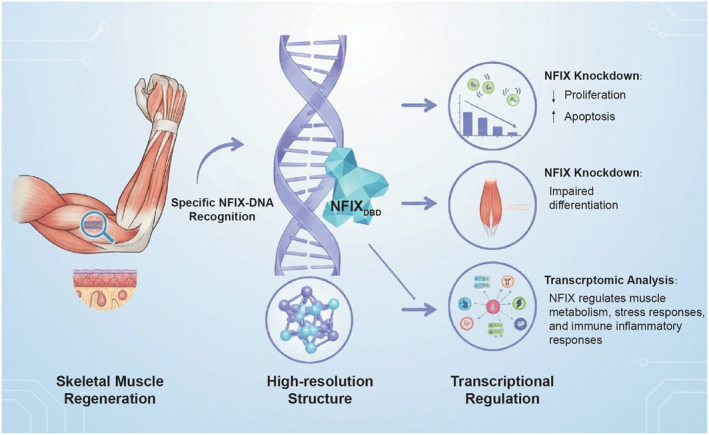

## 1 Introduction

Skeletal muscle is the largest tissue in the human body, accounting for approximately 40% of total body weight and containing 50%–75% of the body's total protein [1]. It plays a vital role in maintaining posture, enabling voluntary movement, and regulating systemic metabolism [2]. Skeletal muscle development is a highly coordinated process involving successive waves of myogenesis, driven by distinct embryonic and fetal myoblast populations with specific gene expression programs and differentiation capacities [3]. These developmental phases establish the structure and functional foundations of muscles, which are essential for postnatal growth and regenerative potential. Dysregulation of these processes can lead to muscle atrophy [4], which is characterized by the progressive loss of muscle mass and strength and is a hallmark of various pathological conditions [5]. These conditions include age‐related sarcopenia [6], cachexia [7], multiple forms of muscular dystrophy [8, 9], and inflammatory myopathies [10]. Such disorders severely impair mobility and quality of life and are closely associated with increased mortality [5, 11, 12]. Therefore, elucidating the molecular mechanisms that govern skeletal muscle maintenance and degeneration is critical for the development of effective therapeutic strategies.

Nuclear Factor I X‐type (NFIX), a member of the NFI transcription factor family, has emerged as a key regulator of skeletal muscle development and homeostasis [13]. During embryonic development, NFIX drives the transition from embryonic to fetal myogenesis by activating fetal‐specific genes and repressing embryonic ones [14, 15]. In adult muscle, NFIX promotes myogenic differentiation and regeneration by inhibiting the expression of myostatin, thereby regulating the timing of satellite cell activation [16, 17]. Furthermore, NFIX cooperates with Sox6 to influence fiber‐type specification, favoring the formation of fast‐twitch muscle fibers [18, 19]. Its expression is modulated by upstream signaling pathways, including the RhoA/ROCK/ERK/JunB axis, and it forms a positive feedback loop with the extracellular matrix protein matrilin‐2 to promote myogenic differentiation [20]. Under pathological conditions, such as muscular dystrophy, NFIX contributes to disease progression by driving aberrant regenerative responses [21]. In myotonic dystrophy type 1, aberrant splicing of NFIX has also been implicated in disease pathogenesis, highlighting its critical role in maintaining muscle homeostasis [22].

**FIGURE 1:**
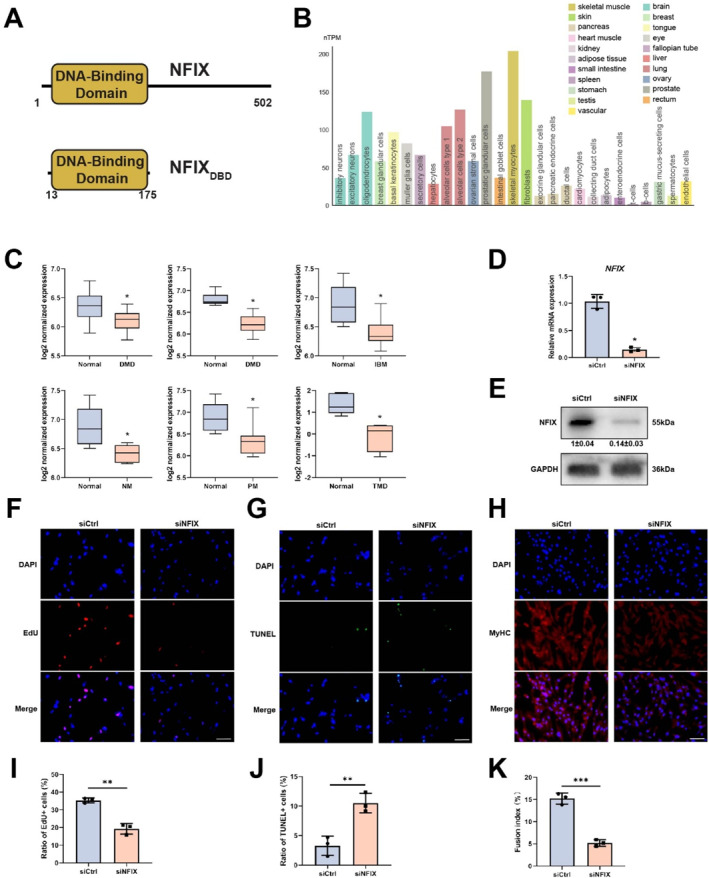
NFIX regulates skeletal muscle cell proliferation, apoptosis, and differentiation. (A) Schematic diagram illustrating the domain architecture of full‐length human NFIX (residues 1–502) and its DNA‐binding domain. (B) Single‐cell RNA‐seq analysis showing *NFIX* expression across various human cell types. (C) *NFIX* expression in skeletal muscle tissues from patients with Duchenne muscular dystrophy (DMD), inclusion body myositis (IBM), nemaline myopathy (NM), polymyositis (PM), and tibial muscular dystrophy (TMD) compared with healthy controls, based on multiple GEO datasets. (D–E) siRNA‐mediated knockdown of NFIX in immortalized human skeletal muscle cells, with depletion efficiency validated by qPCR (D) and Western blot analysis (E). (F, I) EdU incorporation assay showing reduced DNA synthesis in NFIX‐depleted cells compared with control cells (F), quantified as the percentage of EdU‐positive nuclei (I). Scale bar, 100 μm. (G, J) TUNEL assay indicating increased apoptosis in NFIX knockdown cells (G), with quantification of apoptotic nuclei (J). Scale bar, 100 μm. (H, K) Myogenic fusion assay showing reduced myotube formation in NFIX‐deficient cells (H), quantified as fusion index (K). Scale bar, 100 μm. Data in (D–K) are presented as mean ± SD, dots represent individual samples. Analysis by unpaired Student's *t*‐test. For all panels, *n* = 3. *p < 0.05.

Despite its recognized importance in skeletal muscle development, the precise transcriptional networks regulated by NFIX in muscle cells, particularly the molecular mechanisms by which NFIX engages DNA, remain incompletely understood. NFIX is characterized by a conserved N‐terminal DNA‐binding domain (DBD) that mediates sequence‐specific DNA recognition, and a proline‐rich, largely unstructured C‐terminal region subject to alternative splicing and post‐translational modifications, which modulate its transcriptional activity (Figure 1A). Early studies proposed that NFI proteins dimerize through their DBDs to recognize palindromic DNA motifs, such as TGGCA(N_3_)TGCCA [23, 24, 25, 26, 27]; however, subsequent evidence has shown that monomeric binding to TGGCA‐containing half‐sites also plays a biologically relevant role [24, 27, 28]. Nonetheless, direct structural insights into DNA recognition by NFIX remain limited. Although a crystal structure of the human NFIX_DBD_ bound to DNA (PDB ID: 7QQD) has been deposited, it appears biologically irrelevant, as the DNA is positioned externally to the protein and lacks base‐specific interactions. Given the central role of DNA recognition in NFIX‐mediated transcriptional regulation, elucidating its structural and mechanistic basis is essential for understanding its function in skeletal muscle.

To address this gap, we investigated the transcriptional network regulated by NFIX in skeletal muscle cells and elucidated the molecular mechanisms by which NFIX controls gene expression. Using immortalized human skeletal muscle cells, we demonstrated that NFIX knockdown via siRNA impairs cell proliferation, increases apoptosis, and reduces myogenic differentiation capacity, underscoring its essential role in skeletal muscle development. Transcriptomic profiling by RNA sequencing revealed that NFIX regulates a network of genes involved in muscle metabolism, stress responses, and immune inflammation, including direct targets such as *NMNAT2*, *EGR1*, *IL1RN*, and *NDRG2*. To investigate the molecular basis of its DNA recognition, we expressed and purified full‐length NFIX and its DBD. Surprisingly, both NFIX and its DBD exist as monomers in solution. High‐resolution crystal structure of the NFIX_DBD_ bound to palindromic DNA motifs revealed that NFIX engages DNA as a monomer, recognizing a single TGGCA sequence through specific base interactions. These structural findings are supported by biophysical binding assays and cellular luciferase reporter experiments, which confirm that sequence‐specific DNA binding is critical for the transcriptional activation of its target genes. Collectively, our study redefines the mechanism of NFIX–DNA recognition and establishes a structural and functional framework for its role as a transcriptional regulator in skeletal muscle development.

## 2 Results

### 2.1 NFIX Regulates Skeletal Muscle Cell Proliferation, Apoptosis, and Differentiation

NFIX expression is markedly enriched in skeletal myocytes compared to other cell types, suggesting a critical role in skeletal muscle development (Figure 1B). To assess its clinical relevance, we analyzed multiple Gene Expression Omnibus (GEO) datasets that compared *NFIX* expression in skeletal muscle tissues from patients with muscular dystrophies or inflammatory myopathies to that in healthy controls. *NFIX* expression was significantly reduced in Duchenne muscular dystrophy samples across two independent datasets, and was consistently downregulated in tibial muscular dystrophy, inclusion body myositis, nemaline myopathy, and polymyositis patients (Figure 1C). These findings indicate that *NFIX* downregulation is a common feature of diverse myopathic conditions and may contribute to impaired muscle homeostasis and regeneration.

To investigate NFIX function, we employed immortalized human skeletal muscle cells and performed siRNA‐mediated knockdown, with efficient depletion, as confirmed by qPCR (Figure 1D) and Western blot (Figure 1E). EdU incorporation assays revealed a marked reduction in the proportion of EdU‐positive nuclei in NFIX‐deficient cells compared to controls, indicating impaired S phase entry or progression and reduced proliferative capacity (Figure 1F,I). TUNEL assays further demonstrated a substantial increase in apoptosis following *NFIX* knockdown (Figure 1G,J), with quantitative analysis showing more than a 50% increase in apoptotic cells. These results establish NFIX as a key regulator of skeletal muscle cell proliferation and survival. Furthermore, myotube fusion assays showed that NFIX knockdown significantly impaired myotube formation, indicating reduced differentiation potential in NFIX‐deficient cells (Figure 1H,K). Collectively, these results demonstrate that NFIX is essential for skeletal muscle cell proliferation, survival, and differentiation and that its downregulation is a common molecular feature in multiple myopathies, potentially driving compromised tissue homeostasis and defective muscle regeneration.

### 2.2 NFIX‐Mediated Transcriptional Regulation in Skeletal Muscle Cells

**FIGURE 2:**
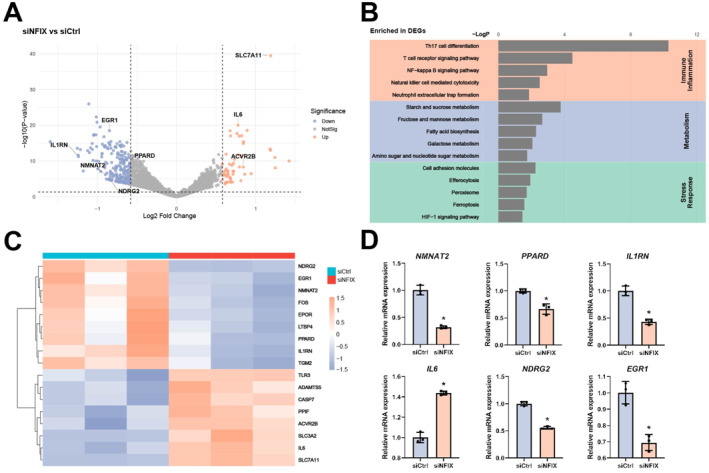
NFIX‐mediated transcriptional regulation in human skeletal muscle cells. (A) Volcano plot showing differentially expressed genes (DEGs) in NFIX knockdown versus control human skeletal muscle cells, as determined by RNA‐seq. Significantly upregulated (red) and downregulated (blue) genes are indicated. (B) KEGG pathway enrichment analysis of DEGs, highlighting significant enrichment in immune and inflammatory responses, metabolic processes, and stress‐related pathways. (C) Heat map illustrating expression changes of representative NFIX‐regulated genes across enriched pathways. (D) qPCR validation of NMNAT2, PPARD, IL1RN, IL6, NDRG2, and EGR1. Data in (D) are presented as mean ± SD, dots represent individual samples. Analysis by unpaired Student's *t*‐test. For all panels, *n* = 3. *p < 0.05.

To explore the mechanisms by which NFIX contributes to muscle homeostasis, we performed RNA sequencing on control and NFIX deficiency human skeletal muscle cells. NFIX knockdown resulted in 247 differentially expressed genes (FDR ≤ 0.05, fold change ≥ 2), including 51 upregulated and 196 downregulated genes (Figure 2A). KEGG pathway enrichment analysis revealed significant involvement of these DEGs in immune and inflammatory responses (e.g., NF‐kappa B signaling pathway), metabolic processes (e.g., fatty acid biosynthesis), and stress‐related pathways (e.g., peroxisome) (Figure 2B). Focusing on key genes within these enriched pathways, volcano plot and heat map analyses showed that NFIX knockdown significantly upregulated the pro‐inflammatory cytokine *IL6* while downregulating the anti‐inflammatory mediator *IL1RN*. We also observed decreased expression of metabolic regulators (*NMNAT2* and *PPARD*) and reduced levels of stress‐response mediators (*NDRG2* and *EGR1*) (Figure 2A,C). qPCR validation confirmed expression changes consistent with the RNA‐seq results (Figure 2D).

**FIGURE 3:**
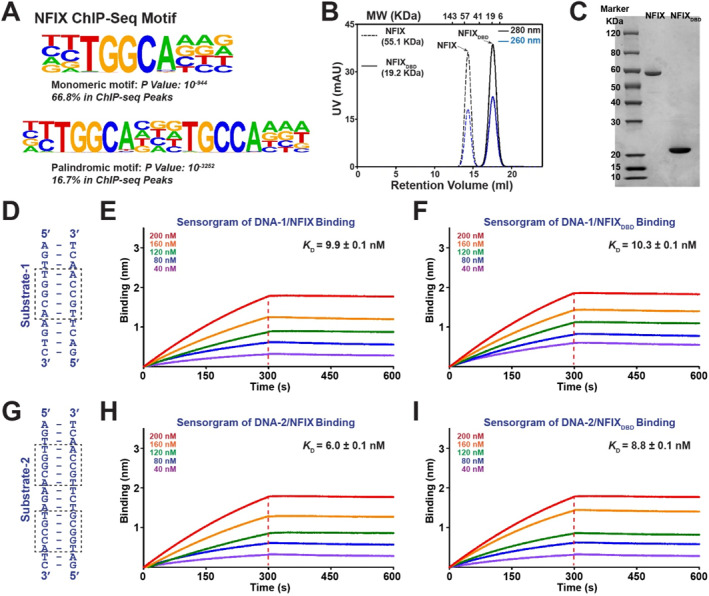
NFIX recognizes TGGCA motifs in a monomeric mode. (A) Motifs enriched in NFIX ChIP‐seq peaks from ENCODE (ENCFF726LLI) identified by HOMER: a TGGCA half‐site (top) and a palindromic dyad (bottom). (B) Size‐exclusion chromatography (Superdex 200 Increase) of full‐length NFIX and the DNA‐binding domain (NFIX_DBD_); elution positions of standards are indicated. (C) Native PAGE analysis confirming the monomeric state of NFIX and NFIX_DBD_ in solution. (D, G) Sequences of biotinylated DNA probes used in binding assays: DNA‐1 (TGGCA half‐site) and DNA‐2 (dyad). (E–F) BLI sensorgrams for NFIX (E) and NFIX_DBD_ (F) binding to DNA‐1; global fits to a 1:1 Langmuir model. (H–I) BLI sensorgrams for NFIX (H) and NFIX_DBD_ (I) binding to DNA‐2 under identical conditions.

To delineate the basis of NFIX DNA recognition in regulating gene expression, we analyzed NFIX ChIP–seq data with HOMER for motif discovery [29]. The TGGCA half‐site was highly enriched, occurring in > 65% of NFIX peaks (Figure 3A), whereas palindromic dyads such as TGGCA(N_3_)TGCCA were also enriched but less frequent. Notably, TGGCA elements were the only NFIX motifs detected in the promoters of validated NFIX targets, including *EGR1* and *NDRG2*, directly linking sequence‐specific binding to transcriptional regulation. Together, these results support a model in which NFIX preferentially engages TGGCA elements within regulatory DNA to drive gene programs relevant to skeletal muscle development and stress responses.

### 2.3 NFIX Binds DNA as a Monomer in Solution

To further elucidate the molecular mechanism underlying NFIX‐mediated gene regulation, we recombinantly expressed and purified both full‐length human NFIX and its isolated DNA‐binding domain for characterization (Figure 1A). While previous models proposed that NFI family members, including NFIX, dimerize through their DBDs to recognize palindromic DNA motifs such as TGGCA(N_3_)TGCCA [23, 24, 30, 31]; however, these conclusions were largely based on experiments using cellular lysates or chemical crosslinking, which may artificially stabilize multimeric complexes and obscure intrinsic oligomeric states under native conditions.

To directly address this, we expressed full‐length NFIX in HEK293 cells and its DBD in *E. coli*, both fused to N‐terminal His_6_‐MBP tags to enhance expression and purification. Following TEV protease cleavage, size‐exclusion chromatography (SEC) revealed that both full‐length NFIX and its DBD eluted as symmetric, monodisperse peaks with retention volumes consistent with monomeric species (Figure 3B). Native PAGE analysis further confirmed that both proteins exist as monomers in solution (Figure 3C), challenging the long‐standing assumption that NFI proteins dimerize via their DBDs. These findings demonstrate that NFIX remains monomeric under physiological conditions, both in its full‐length and DBD‐only forms.

We next investigated the DNA‐binding properties of NFIX using biolayer interferometry (BLI) with biotinylated DNA probes corresponding to canonical NFIX recognition motifs: a 12‐bp TGGCA monomeric site (DNA‐1) and an 18‐bp palindromic dyad (DNA‐2) (Figure 3D,G). Both DNAs exhibited high‐affinity binding to full‐length NFIX with dissociation constants of 9.9 and 6.0 nM, respectively (Figure 3E,H, Supporting Information S1: Figure S1). Binding occurred with 1:1 stoichiometry, consistent with monomeric DNA engagement. Similar results were observed with isolated DBD (Figure 3F,I, Supporting Information S1: Figure S1, Table S1), confirming that the DBD is sufficient for specific recognition of both motif types. Taken together, these results demonstrate that NFIX binds the tested DNA substrates as a monomer through its DBD, providing a revised mechanistic framework for understanding NFIX's sequence‐specific gene regulation.

### 2.4 Structure of NFIX Bound to Palindromic DNA

**FIGURE 4:**
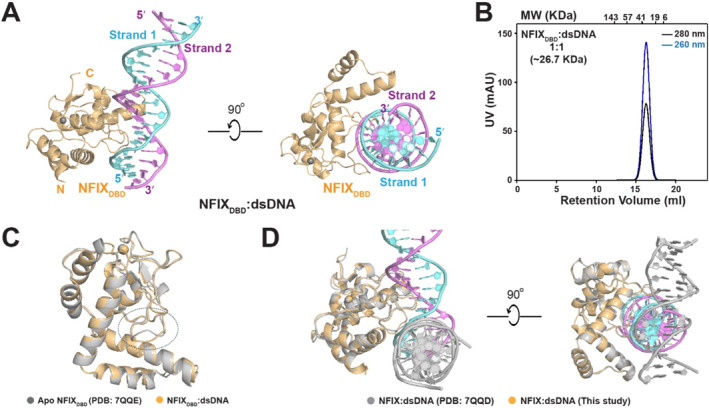
Crystal structure of NFIX_DBD_ bound to a palindromic duplex. (A) Overall structure of NFIX_DBD_ (orange) in complex with an 18‐bp dsDNA (strand 1, cyan; strand 2, magenta). Orthogonal views depict major‐groove engagement and the binding orientation along one face of the helix. (B) Size‐exclusion chromatography of the purified complex shows a single symmetric peak consistent with a 1:1 NFIX_DBD_:DNA stoichiometry. (C) Superposition with the apo NFIX_DBD_ structure (PDB 7QQE) reveals a ligand‐induced movement of the DNA‐binding loop. (D) Comparison with the previously deposited NFIX:dsDNA structure (PDB 7QQD) indicates that the present structure captures a specific, productive binding mode, whereas 7QQD places the DNA without base‐specific contacts.

Although NFIX has been extensively studied for its biological functions, the structural basis of its DNA recognition has remained unresolved. To address this issue, we determined the crystal structure of the NFIX DNA‐binding domain in complex with a palindromic DNA duplex containing the canonical TGGCA(N_3_)TGCCA motif (Figure 3G), historically proposed to mediate dimeric binding by NFI family proteins. Crystals of the NFIX_DBD_:dsDNA complex diffracted to 2.3 Å resolution (Figure 4A, Supporting Information S1: Table S2). The structure revealed a single NFIX_DBD_ molecule bound to one end of a B‐form DNA helix with a clear 1:1 protein–DNA stoichiometry, consistent with SEC and BLI data (Figure 4B, Supporting Information S1: Figure S7). The asymmetric unit contains one NFIX polypeptide (residues 13–171), two complementary DNA strands, and a coordinated zinc ion essential for structural stability. NFIX engages the DNA major groove via a loop insertion that induces localized helical deformation without disrupting the overall duplex architecture. The extensive interface (∼805 Å^2^ buried surface area) supports high‐affinity, sequence‐specific binding.

Two NFIX crystal structures have been deposited in the Protein Data Bank (PDB IDs: 7QQD and 7QQE) [32]. The apo NFIX structure (7QQE, 3.5 Å) depicts the unbound conformation, and comparison with our DNA‐bound structure revealed localized conformational adjustments in the loop region upon DNA binding, while the overall fold remains conserved (Figure 4C). The previously deposited NFIX–DNA structure (7QQD, 2.7 Å) positions the DNA peripherally, lacking base‐specific contacts or proper groove engagement (Figure 4D), rendering it biologically implausible. Thus, our structure provides the first functionally validated, high‐resolution view of NFIX–DNA recognition, resolving longstanding uncertainties and establishing a mechanistic framework for its sequence‐specific binding.

### 2.5 Molecular Mechanism of dsDNA Recognition by NFIX

**FIGURE 5:**
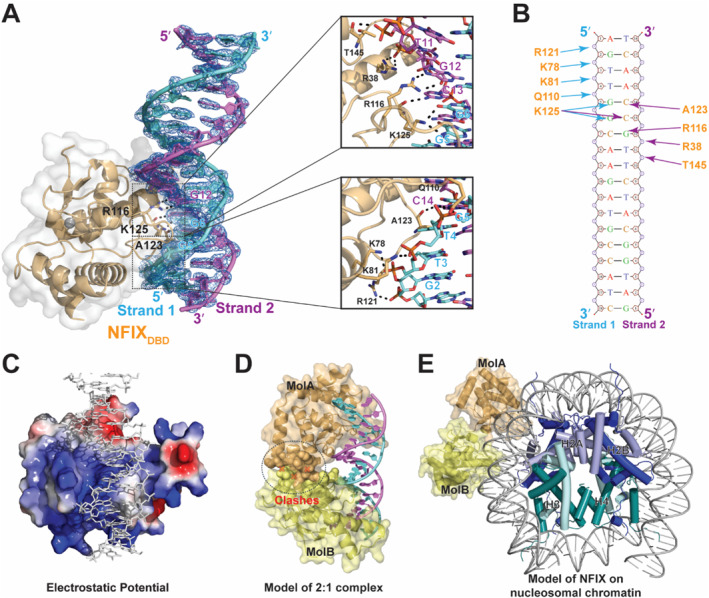
Structural basis of sequence‐specific DNA recognition by NFIX. (A) Detailed interactions of the NFIX_DBD_:dsDNA interface. NFIX_DBD_ is shown as a ribbon with a semitransparent surface; the duplex is contoured with a composite‐omit 2m*F*_o_–D*F*_c_ electron density map contoured at 2*σ*. Insets highlight base‐specific contacts and phosphate‐backbone interactions formed by key residues. (B) Schematic diagram summarizing the protein–DNA contacts observed in the NFIX_DBD_:dsDNA structure. (C) Electrostatic potential surface of NFIX_DBD_ at the DNA interface, illustrating the basic patch complementary to the DNA backbone. (D) Model of a hypothetical 2:1 NFIX_DBD_:dsDNA assembly generated by aligning two NFIX molecules to the symmetric half‐sites. Extensive steric clashes (indicated) argue against simultaneous dimeric occupancy of the palindromic motif on short B‐form dsDNA. (E) Model of a hypothetical NFIX dimer on nucleosomal chromatin at a dyad motif. Bending of nucleosomal DNA permits simultaneous binding of two NFIX molecules.

The NFIX_DBD_:dsDNA structure reveals a distinct mode of DNA recognition, combining phosphate‐backbone interactions with precise base‐specific recognition of the TGGCA motif. Conserved basic and polar residues, R38, K78, K81, Q110, and R121, form a network of electrostatic contacts with the backbone phosphates of both DNA strands (Figure 5A, Supporting Information S1: Table S3). Sequence‐specific readout is mediated by R116, A123, and K125: R116 donates two hydrogen bonds to guanine G12 on strand 2; the backbone carbonyl of A123 hydrogen bonds with cytosine C14; and K125 contacts guanines G5 and G6 on strand 1 as well as cytosine C13 on the complementary strand (Figure 5B). These base‐specific interactions are embedded within a continuous, positively charged surface spanning both strands, effectively bridging the major groove and anchoring NFIX at its cognate site (Figure 5C). This represents the atomic‐resolution depiction of NFIX–DNA recognition, establishing a structural framework for its sequence‐specific transcriptional regulation.

To test whether NFIX can simultaneously engage both half‐sites of the palindromic dyad, we modeled a complex containing two NFIX_DBD_ molecules bound to opposite ends of the motif. The model revealed severe steric clashes arising from the alignment of major grooves on the same face of the DNA helix (Figure 5D), rendering dual occupancy structurally prohibitive. Consistently, biolayer interferometry confirmed a strict 1:1 protein–DNA stoichiometry. Together, these structural and biophysical data support a prevalent monomeric binding mode, indicating that NFIX recognizes palindromic motifs as a monomer under our conditions.

### 2.6 Specific DNA Binding Is Indispensable for NFIX‐Mediated Transcription Regulation

**FIGURE 6:**
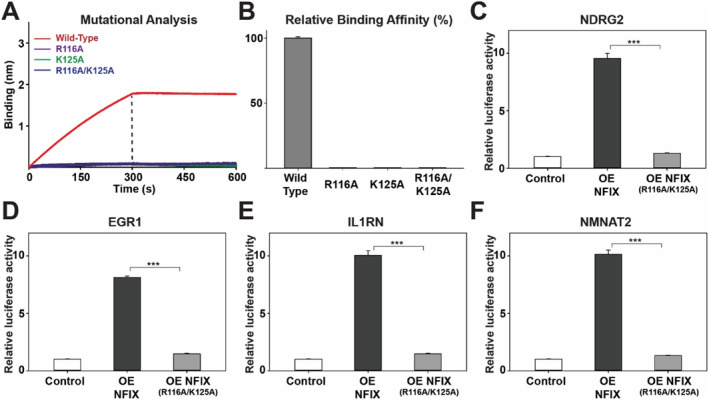
NFIX activates skeletal muscle–related promoters via specific DNA recognition. (A) Mutational analysis of sequence‐recognition residues in NFIX_DBD_ using BLI, comparing the dsDNA binding profiles of wild‐type NFIX_DBD_ with R116A, K125A, and R116A/K125A mutants. (B) Quantification of relative DNA‐binding affinities, calculated as WT *K*_D_ ÷ mutant *K*_D_ × 100. (C–F) Dual‐luciferase assays with pGL3 reporters driven by the *NDRG2*, *EGR1*, *IL1RN*, and *NMNAT2* promoters. WT NFIX robustly enhances reporter activity, whereas the DNA‐binding–defective double mutant (R116A/K125A) fails to activate transcription. Data are mean ± s.d. from ≥ 3 independent experiments; unpaired two‐tailed *t*‐test: ***p < 0.001.

To directly test the functional relevance of the DNA contacts identified in our structure, we performed site‐directed mutagenesis combined with BLI binding assays. Structural analysis pinpointed three residues, R116, A123, and K125, as critical for sequence‐specific recognition of the TGGCA motif: R116 and K125 engage in base‐specific side‐chain contacts, while the backbone carbonyl of A123 hydrogen bonds with cytosine C14. We generated single (R116A, K125A) and double (R116A/K125A) alanine mutants of NFIX_DBD_ (Supporting Information S1: Figure S5). Whereas wild‐type NFIX_DBD_ bound the palindromic motif with high affinity, all mutant variants exhibited a near‐complete loss of binding, with negligible BLI wavelength shifts (Figure 6A) and > 99% reduction in relative affinity (Figure 6B).

We next assessed whether sequence‐specific DNA binding is essential for NFIX‐dependent transcriptional activation. Promoter regions of four NFIX target genes—*NDRG2*, *EGR1*, *IL1RN* and *NMNAT2*—identified from our RNA‐seq dataset were cloned upstream of luciferase reporters, each retaining the intact wild‐type TGGCA motif. Overexpression of wild‐type NFIX robustly activated all promoters (Figure 6C–F). In contrast, expression of the R116A/K125A mutant completely abolished promoter activation despite the presence of the unaltered recognition motif (Figure 6C–F). These results validate the structural model of NFIX–DNA recognition and provide direct functional evidence that sequence‐specific DNA binding is essential for NFIX‐driven transcriptional programs. Together, they establish a mechanistic link between NFIX's cognate motif recognition and its transcriptional regulation of genes involved in skeletal muscle development and stress response.

## 3 Discussion

Transcription factors (TFs) orchestrate complex gene expression programs that govern development, tissue homeostasis, and adaptive responses to disease [33, 34]. In skeletal muscle, we identify NFIX as a central transcriptional regulator that promotes myoblast proliferation while suppressing apoptosis and premature differentiation. Transcriptomic profiling indicates that NFIX governs a regulatory network enriched in muscle‐related pathways, with representative targets including *NDRG2*, *EGR1*, *IL1RN*, and *NMNAT2*, each containing conserved NFI‐binding motifs within their promoter regions. Functionally, disruption of key DNA‐recognition residues abolishes NFIX‐mediated transcriptional activation, underscoring the essentiality of sequence‐specific DNA binding for its regulatory activity.

Clinically, NFIX expression is strongly associated with skeletal muscle health. Analysis of human skeletal muscle transcriptomes revealed significant downregulation of NFIX expression in Duchenne muscular dystrophy and several other myopathies. Notably, these human data contrast with a murine study in which *Nfix* ablation alleviated pathological features in a mouse model of muscular dystrophy [21], suggesting species‐specific and context‐dependent roles for NFIX. Such discrepancies underscore the necessity of dissecting NFIX's mechanistic functions across different disease stages and biological systems, and highlight its potential as a therapeutic target in muscle disorders.

To elucidate such a mechanism of NFIX function, we determined the high‐resolution crystal structure of its DNA‐binding domain bound to a palindromic dyad‐symmetric DNA sequence [TGGCA(N_3_)TGCCA]. The structure revealed that NFIX engages DNA as a monomer through a conserved major groove recognition mechanism. Base‐specific interactions are mediated by R116, A123, and K125, residues conserved across the NFI family, suggesting a shared recognition mode among family members (Supporting Information S1: Figure S2). Although the JASPAR database groups the NFI DBD with SMAD MH1 domains, our structural comparisons demonstrate substantial differences. The NFI DBD features an extended *α*‐helical region for DNA engagement, whereas the MH1 domain relies on a *β*‐hairpin motif (Supporting Information S1: Figure S3). Furthermore, sequence specificity differs markedly: NFIX recognizes the TGGCA motif, whereas SMAD MH1 domains target CAGAC [35]. These distinctions establish NFI proteins as a unique class of DNA‐binding domains with a previously underappreciated recognition mechanism.

To further evaluate the novelty of this binding mode, we used AlphaFold3 to predict NFIX_DBD_:dsDNA complexes. However, the predicted models exhibited low confidence, failed to reproduce the experimentally observed protein–DNA contacts, and instead generated an entirely inverted DNA orientation (Supporting Information S1: Figure S4). These discrepancies underscore that the interactions revealed in our crystal structure represent a previously uncharacterized mode of protein–DNA recognition not captured by existing structures.

Our findings refine the DNA‐recognition model for NFI proteins. Despite the dyad‐symmetric motif, our 2.3 Å structure on a short, linear B‐form DNA substrate, and solution assays indicate a prevalent monomeric 1:1 mode under our conditions, in which two NFIX molecules cannot be accommodated simultaneously without spatial conflict. By contrast, a recent bioRxiv study on mouse NFIX [32], using cryo‐EM under detergent conditions with longer DNA, reported apparent dimeric engagement within a helical filament assembly at a palindromic site, suggesting that DNA geometry and experimental milieu can favor alternative assemblies. Given the significant enrichment of dyad motifs in our ChIP‐seq analysis (Figure 3A), we hypothesize that dimeric binding may occur in chromatin, where bent or nucleosome‐constrained DNA and cofactor scaffolds can enable two‐site occupancy. Consistent with this context‐dependent view, a chromatin‐mimetic model accommodates two NFIX_DBD_ molecules on a palindromic site by widening the binding geometry (Figure 5E, Supporting Information S1: Figure S6). Future work using nucleosome‐reconstituted templates and targeted cofactor perturbations will be needed to define the prevalence and regulatory impact of such dimeric configurations.

Together, by integrating transcriptomic, structural, and functional evidence, our study provides the first functionally validated, atomic‐resolution model of NFIX–DNA recognition. This framework offers a rational basis for developing structure‐guided strategies to modulate NFIX activity, with potential applications in the treatment of muscular dystrophies, inflammatory myopathies, and other NFIX‐associated disorders, thereby opening new avenues for translational research in muscle biology and disease intervention.

## 4 Materials and Methods

### 4.1 Single‐Cell nTPM Quantification for NFIX

The single‐cell RNA sequencing data for the NFIX gene were obtained from the Human Protein Atlas (HPA) single‐cell database (gene ID: ENSG00000008441). Tissue datasets and cluster data were downloaded from the database (https://www.proteinatlas.org/humanproteome/single+cell/single+cell+type/data#datasets) to retrieve the gene expression levels of NFIX in the target tissues and cell types. The expression value for each cell type was generated by calculating the weighted average nTPM of all cells within the same cluster annotation in a given tissue.

### 4.2 Gene Expression Microarray Analysis From GEO

Microarray datasets related to muscle diseases were obtained from the Gene Expression Omnibus (GEO) database (https://www.ncbi.nlm.nih.gov/geo/). The selected datasets included samples from patients with Duchenne muscular dystrophy (GSE6011 and GSE38417), tibial muscular dystrophy (GSE42806), and inflammatory myopathies—including necrotizing myopathy, polymyositis, and inclusion body myositis (GSE39454). All datasets were derived from skeletal muscle biopsy samples collected from clinically diagnosed patients and included matched healthy controls.

Raw or preprocessed expression data were retrieved and log2‐transformed if necessary. Probe identifiers were mapped to gene symbols based on the corresponding platform annotation files. In cases where multiple probes corresponded to the same gene, the probe with the highest average expression across all samples was retained.

To assess the disease‐associated expression pattern of *NFIX*, we performed single‐gene differential expression analysis between disease and control groups. Expression values were compared using a two‐tailed unpaired *t*‐test with Welch's correction to account for unequal variances and sample sizes. A *p*‐value < 0.05 was considered statistically significant. All analyses were performed using R (version 4.5.1).

### 4.3 Cell Culture

Human immortalized skeletal muscle cells (Cellverse, #iCell‐0086a) were cultured in a specialized growth medium for immortalized human skeletal muscle cells (Cellverse, #iCell‐0086a‐001b) under standard conditions at 37°C in a humidified atmosphere containing 5% CO_2_. Cells were passaged when they reached 70%–80% confluence. For gene knockdown experiments, cells were seeded in 12‐well plates at 50% confluence. Transfection was performed using RNATransMate reagent (Sangon Biotech, #E607402) with 5 nM small interfering RNA (siRNA) targeting NFIX (Sangon Biotech) or non‐targeting control siRNA (Sangon Biotech), following the manufacturer's instructions. Cells were harvested 36 h post‐transfection for subsequent analyses.

### 4.4 Quantitative Real‐Time PCR (qRT‐PCR)

Total RNA was extracted from all samples using the EZ‐press RNA Purification Kit (EZB, #B0004D) according to the manufacturer's protocol. RNA concentration and purity were verified spectrophotometrically (A260/A280 ratio). Genomic DNA elimination and reverse transcription were carried out using the EZscript All‐in‐One Reverse Transcription Kit (with DNase) (EZB, #RT3) under specified reaction conditions. Synthesized cDNA served as a template for real‐time PCR amplification using gene‐specific primers and probes. Amplification reactions were conducted with the following thermal cycling parameters: initial denaturation at 95°C for 30 s, followed by 40 cycles of 95°C for 5 s and 60°C for 30 s. Fluorescence data were collected during the annealing/extension phase. Finally, the relative expression of the target genes was calculated by the 2‐ΔΔCT method [36], with GAPDH serving as an internal reference. The relevant primer sequences are shown in Supporting Information S1: Table S4. All reactions included no‐template controls and were performed in technical triplicate.

### 4.5 Western Blot

Protein samples were extracted from human skeletal muscle cells using RIPA lysis buffer (Beyotime, #P0013B). Protein concentrations were determined using a BCA protein assay kit (Beyotime, #P0010). Equal amounts of protein (20 μg) were separated by SDS–PAGE and transferred onto PVDF membranes (Millipore, Billerica). The membranes were incubated overnight at 4°C with the primary antibody, followed by a 1‐h incubation with the secondary antibody. Protein bands were visualized using the ChemiDOC Western blot imaging system (BIO‐RAD). The primary antibody used for Western blotting was NFIX (Proteintech, #67983‐1‐Ig, 1:1000), with Tubulin (Proteintech, #66031‐1‐Ig, 1:1000) serving as the loading control.

### 4.6 EdU Incorporation Assay

Cell proliferation was assessed using an EdU incorporation assay kit (Beyotime, #C0078S) according to the manufacturer's instructions. Human skeletal muscle cells were seeded into 35‐mm glass‐bottom dishes (Biosharp, #BS‐20‐GJM). Following siRNA transfection, cells were fixed with 4% paraformaldehyde and incubated with EdU to allow incorporation into newly synthesized DNA during active DNA replication. Fluorescently labeled cells were subsequently imaged and analyzed using a confocal laser scanning microscope (TCS SP8, Leica Microsystems). Each condition was performed in three biological replicates.

### 4.7 TUNEL Assay

Apoptotic cells were detected using the DeadEnd Fluorometric TUNEL System (Promega, #G3250) according to the manufacturer's instructions. Human skeletal muscle cells were seeded in a 35‐mm glass‐bottom dish. After siRNA transfection, cells were fixed with 4% paraformaldehyde and permeabilized with 0.3% Triton X‐100. The cells were then incubated with the TUNEL reaction mixture to label fragmented DNA ends characteristic of apoptosis. Fluorescent signals were visualized and quantified using a confocal laser scanning microscope. Each condition was performed in three biological replicates.

### 4.8 Myotube Fusion Assay

To evaluate myogenic fusion, human skeletal muscle cells were seeded in a 35‐mm glass‐bottom dish. At the time of transfection with *NFIX*‐targeting or control siRNA, the growth medium was simultaneously replaced with differentiation medium consisting of Dulbecco's Modified Eagle Medium (DMEM, Servicebio, #G4515) supplemented with 2% horse serum (Punosai, #164215) to induce myotube formation.

After 72 h of differentiation, cells were fixed with 4% paraformaldehyde, and permeabilized with 0.1% Triton X‐100. Following PBS washes, nonspecific binding was blocked with 5% bovine serum albumin (BSA, Sigma, #A4737) for 1 h at room temperature. Subsequently, cells were incubated overnight at 4°C with an anti‐Myosin Heavy Chain (MyHC) primary antibody (1:500, Proteintech, #22281‐1‐AP), a marker of myogenic differentiation. Fluorescent secondary antibody (1:500, Proteintech, #SA00013‐2) was applied for 1 h at room temperature, followed by counterstaining with DAPI. Images were captured using a confocal laser scanning microscope. Fusion efficiency was quantified using ImageJ software by calculating the fusion index, defined as the percentage of nuclei located within MyHC‐positive multinucleated myotubes. Each condition was performed in three biological replicates.

### 4.9 RNA‐Seq Analysis

Total RNA was extracted using TRIzol Reagent (TIANGEN, #DP424). The concentration, purity, and integrity of RNA were assessed using a NanoDrop spectrophotometer (Thermo Scientific). For each condition, three biological replicates (independent transfections and RNA isolations) were processed. For each replicate, 3 μg of RNA were used as input material for the RNA sample preparations. Sequencing libraries were generated according to the following steps. Firstly, mRNA was purified from total RNA using poly‐T oligo‐attached magnetic beads. Fragmentation was carried out using divalent cations under elevated temperature in an Illumina proprietary fragmentation buffer. First strand cDNA was synthesized using random oligonucleotides and Super Script II. Second strand cDNA synthesis was subsequently performed using DNA Polymerase I and RNase H. The remaining overhangs were converted into blunt ends via exonuclease/polymerase activities and the enzymes were removed. After adenylation of the 3′ ends of the DNA fragments, Illumina PE adapter oligonucleotides were ligated to prepare for hybridization. To select cDNA fragments of the preferred 400–500 bp in length, the library fragments were purified using the AMPure XP system (Beckman Coulter). DNA fragments with ligated adapter molecules on both ends were selectively enriched using Illumina PCR Primer Cocktail in a 15‐cycle PCR reaction. Products were purified using the AMPure XP system (Beckman Coulter) and quantified using the Agilent high sensitivity DNA assay on a Bioanalyzer 2100 system (Agilent Technologies). The sequencing libraries were then sequenced on the NovaSeq 6000 platform (Illumina) at Genekinder Medicaltech (Shanghai) Co. Ltd., China. Differential gene expression analysis was conducted using the DESeq2 R package (v1.48.1) [37]. Genes meeting FDR ≤ 0.05 and fold change ≥ 2 were considered significantly differentially expressed.

### 4.10 ChIP‐Seq Data Analysis

NFIX ChIP‐Seq data (accession: ENCFF726LLI) were retrieved from the ENCODE database. These filtered and aligned binding peaks were subsequently examined using *findMotifsGenome.pl* for motif discovery and *annotatePeaks.pl* for genomic annotation and quantification. Palindromic sites were annotated first, and TGGCA half‐sites were then quantified after excluding dyads using *annotatePeaks.pl* ‐fm filter. Enriched NFIX‐binding motifs were identified, and motif logos were generated using WebLogo for visualization [38].

### 4.11 Expression and Purification of Human NFIX and Its DBD

The cDNA encoding full‐length human NFIX was synthesized and cloned into the pcDNA3.1 vector with an N‐terminal His_6_‐MBP tag followed by a TEV protease cleavage site. Protein expression was carried out using the Expi293 expression system (Thermo Fisher Scientific). Five days post‐transfection, cells were harvested by centrifugation and stored at −80°C until purification.

The NFIX DNA‐binding domain (residues 13–175) was cloned into the pMAL‐c5X vector containing an N‐terminal His_6_‐MBP tag and a TEV cleavage site. The construct was overexpressed in *Escherichia coli* BL21 (DE3) (Beyotime, #D1013). Cultures were grown in LB medium at 37°C to mid‐log phase, induced with 1 mM IPTG, and incubated for an additional 16 h at 18°C. Cells were collected by centrifugation and stored at −80°C.

Both full‐length NFIX and the DBD were purified using an ÄKTA system (GE Healthcare) at room temperature. Cell pellets were resuspended in lysis buffer [30 mM Tris‐HCl (pH 7.5), 200 mM NaCl, 10% glycerol, 1 mM TCEP, 25 mM imidazole], lysed by sonication, and clarified by centrifugation at 26,916 × g for 30 min. The supernatant was loaded onto a 5 mL HisTrap FF column pre‐equilibrated with lysis buffer, and bound proteins were eluted with elution buffer containing 300 mM imidazole. Eluted fractions were incubated overnight with 0.2 mg/mL TEV protease. The digested sample was buffer‐exchanged into lysis buffer and re‐applied to a HisTrap column to remove the uncleaved fusion protein and TEV. The flow‐through containing cleaved NFIB was concentrated and further purified by gel filtration using a Superdex 75 column equilibrated in SEC buffer [25 mM Tris‐HCl (pH 7.5), 200 mM NaCl, 1 mM TCEP]. Peak fractions were collected and assessed by SDS‐PAGE for purity.

### 4.12 Native PAGE Analysis

Native PAGE was performed on precast 4%–20% polyacrylamide gels (Solarbio, #PG42010‐N) using SDS‐free running buffer. Protein samples were mixed with 5× native loading buffer (Sangon Biotech, #C506032) and kept on ice before running. Gels and buffer were pre‐chilled, and electrophoresis was carried out at 4°C and 120 V for 45 min. After electrophoresis, the gels were stained with Coomassie Brilliant Blue G‐250 (Yeasen, #20308ES72) to visualize bands.

### 4.13 Biolayer Interferometry Binding Assay

DNA‐binding kinetics of wild‐type NFIX and its mutant variants were quantitatively assessed using biolayer interferometry on an Octet K2 system (ForteBio). Biotinylated single‐stranded oligonucleotides and their complementary strands were synthesized (General Biol), annealed to form double‐stranded probes, and immobilized onto streptavidin (SA) biosensors. The probe sequences used were 5′‐Biotin‐AGTTGGCAAGTC‐3′ paired with 5′‐GACTTGCCAACT‐3′ (designated DNA‐1), and 5′‐Biotin‐AGTTGGCAAGATGCCATC‐3′ paired with 5′‐GATGGCATCTTGCCAACT‐3′ (DNA‐2). DNA loading was performed at concentrations of 1 ng/μL in assay buffer [20 mM Tris‐HCl (pH 7.5), 100 mM NaCl, 1 mM ZnCl_2_]. Following probe immobilization to saturation, the biosensors were exposed to graded concentrations of NFIX proteins in the same buffer. Association and dissociation phases were recorded at 30°C, and binding curves were analyzed using the ForteBio data analysis software with a 1:1 binding model to determine relative binding affinities.

### 4.14 Crystal Structure Determination

The purified NFIX DNA‐binding domain was concentrated to 10 mg/mL in SEC buffer and used immediately for crystallization trials. For complex formation, synthetic oligonucleotides corresponding to DNA‐2 were purchased from General Biol and annealed using standard thermal cycling protocols. NFIX_DBD_ was mixed with DNA‐2 and incubated on ice for 30 min prior to crystallization. Crystals of the NFIX_DBD_:dsDNA complex were obtained using a reservoir solution containing 0.04 M potassium phosphate monobasic, 16% (w/v) PEG 8000%, and 20% (v/v) glycerol. For cryoprotection, crystals were briefly soaked in reservoir solution supplemented with 20% (v/v) ethylene glycol and flash‐frozen in liquid nitrogen.

X‐ray diffraction data were collected at −173°C with a wavelength of 0.9795 Å on beamline BL18U1 at the Shanghai Synchrotron Radiation Facility (SSRF), using a PILATUS3 S 6M detector. Data were processed with HKL‐3000 [39]. Structures of the NFIX_DBD_:dsDNA complexes were determined by molecular replacement using PHASER [40], with the PDB 7QQE as the search template, and DNA coordinates were built de novo into the electron density map using Coot [41]. Iterative rounds of model building and refinement were carried out using Coot and Phenix [42]. Final structural models were validated via the wwPDB validation server [43]. Data collection and refinement statistics are summarized in Supporting Information S1: Table S2.

### 4.15 Dual‐Luciferase Reporter Assay

Transcriptional activation by NFIX was quantified using a dual‐luciferase assay in HEK293 cells (Guangzhou Yuanjing Biotechnology, #YKO‐HA229822). Cells were plated in 24‐well plates to reach ∼60% confluence at transfection. Promoter fragments (∼2 kb upstream of the TSS) from human *NDRG2*, *EGR1*, *IL1RN*, and *NMNAT2* were cloned into pGL3‐Basic (firefly luciferase; Servicebio). Cells were co‐transfected with expression plasmids encoding wild‐type NFIX, R116A/K125A mutant, or empty pcDNA3.1 (vector control) using Lipofectamine 3000 (Thermo Fisher Scientific), together with pRL‐TK (Renilla luciferase; Promega) for normalization. Each condition was performed in biological triplicate. At 48 h post‐transfection, cells were lysed and luminescence was measured with the Dual‐Luciferase Reporter Assay System (Yeasen) following the manufacturer's protocol. Firefly signals were normalized to Renilla to correct for transfection efficiency. HEK293 cells were routinely authenticated and confirmed to be mycoplasma negative prior to use.

## Author Contributions

J.S. and H.S. conceived and designed the study. C.Z., Xi C., and Y.W. prepared protein samples. C.Z. performed binding assays and analyzed the resulting data. C.Z., Xi C., C.Q., Xin C., and H.S. carried out X‐ray crystallography experiments and subsequent structural analyses. S.L., C.X., L.L., and W.D. conducted cellular assays, bioinformatic analyses, and ChIP‐Seq/RNA‐Seq analyses. X.L., J.S., and H.S. supervised the research. J.S. and H.S. wrote the manuscript with input from all authors.

## Ethics Statement

This study did not include new data from human participants or animals and was exempt from ethical approval.

## Conflicts of Interest

The authors declare no conflicts of interest.

## Supporting information


Supporting Information S1

## Data Availability

Atomic coordinates and structure factors have been deposited in the Protein Data Bank under accession code 9WA7. RNA‐seq data are available in GEO under accession GSE305570. All other data supporting the findings of this study are provided in the paper and its supplementary information or are available from the corresponding author upon reasonable request.
