# Supplementary material for: Mechanistic Insights Into NFIX‐Mediated DNA Recognition and Transcriptional Regulation in Skeletal Muscle
Source: Smart Med. 2026 Jan 29;5(1):e70027. doi: 10.1002/smmd.70027 (PMC12854730; doi:10.1002/smmd.70027)
Supplement: Supplementary file 1 — Supporting Information S1 [file SMMD-5-e70027-s001.pdf]

Supporting Information

Mechanistic Insights into NFIX-Mediated DNA Recognition and Transcriptional Regulation in Skeletal Muscle

Ci Zhu, Shuang Liu, Xi Chen, Chengxiao Qin, Yueyu Wang, Chunchun Xue, Lingxing Li, Wenlan Du, Xin Chen, Xiaofeng Li, Jie Shen*, and He Song**

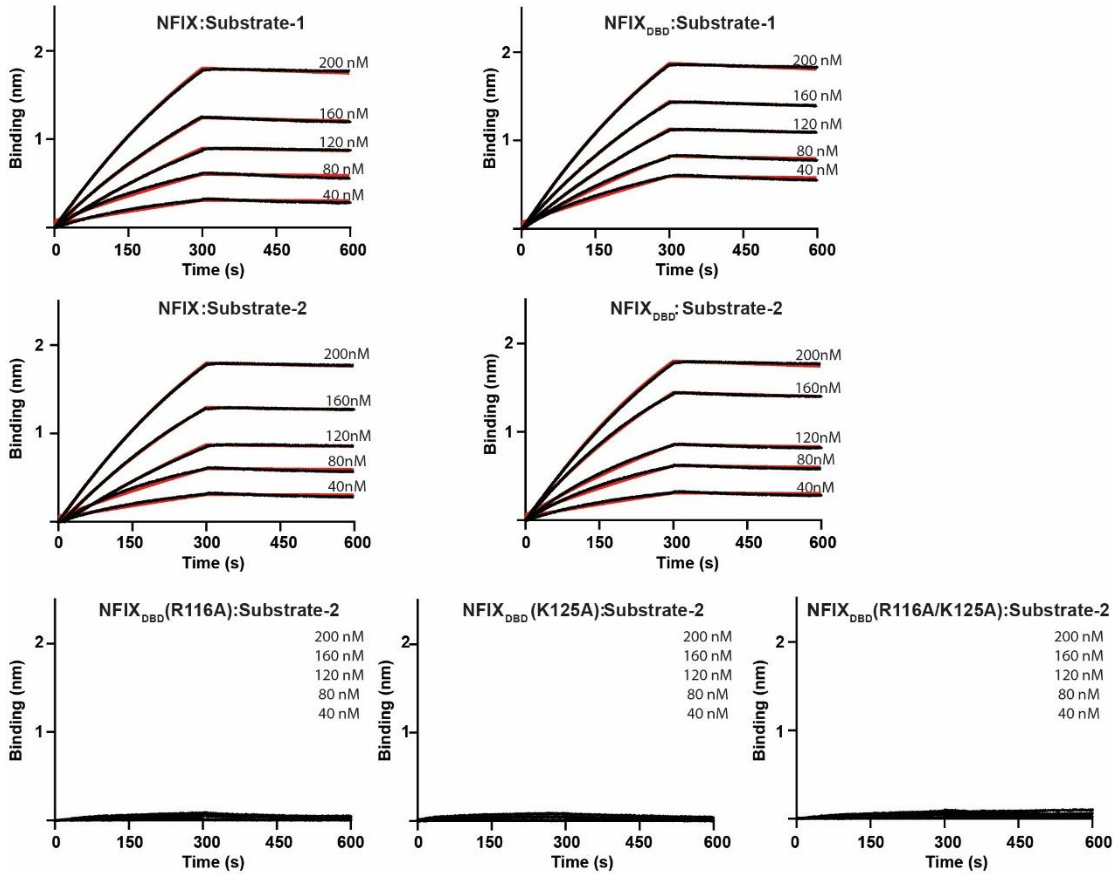

Figure S1. BLI binding characterization of NFIX and its mutants. Sensorgrams with global fits for binding of full-length NFIX, NFIX$_{DBD}$, and point mutants (R116A, K125A, R116A/K125A) to two DNA substrates. SA biosensors bearing biotinylated DNA were exposed to serial protein dilutions (200–40 nM). Kinetic constants are reported in Supplementary Table S1.

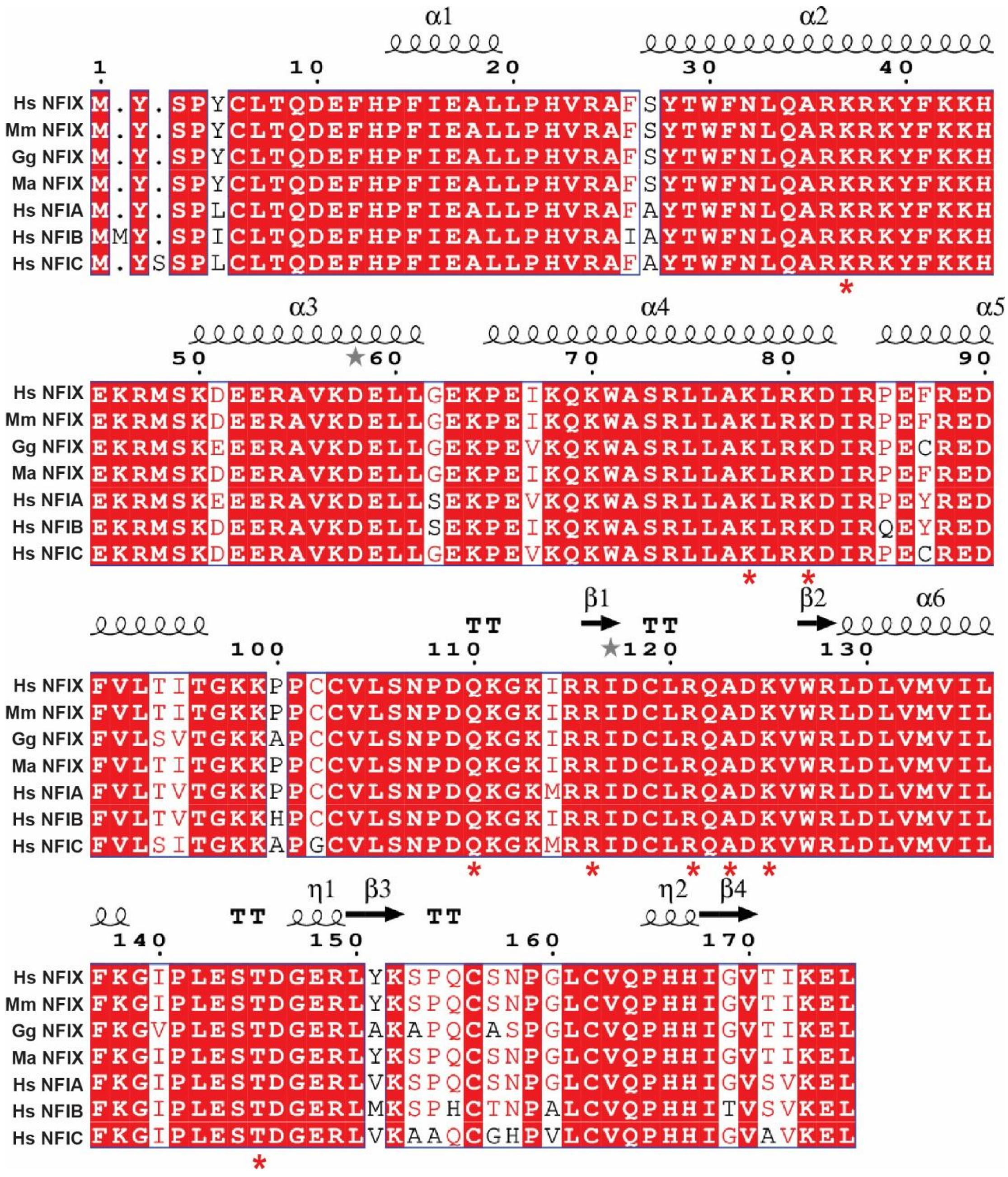

Figure S2. Comparison of the DBD of NFIX family proteins with other NFIs. Multiple sequence alignment of DNA-binding domains from *H. sapiens* NFIX (Q14938), *M. musculus* NFIX (P70257), *G. gallus* NFIX (Q90932), and *M. auratus* NFIX (P13623), alongside *H. sapiens* NFIA (Q12857), NFIB (O00712), and NFIC (P08651). DNA-contacting residues are marked with asterisks (*).

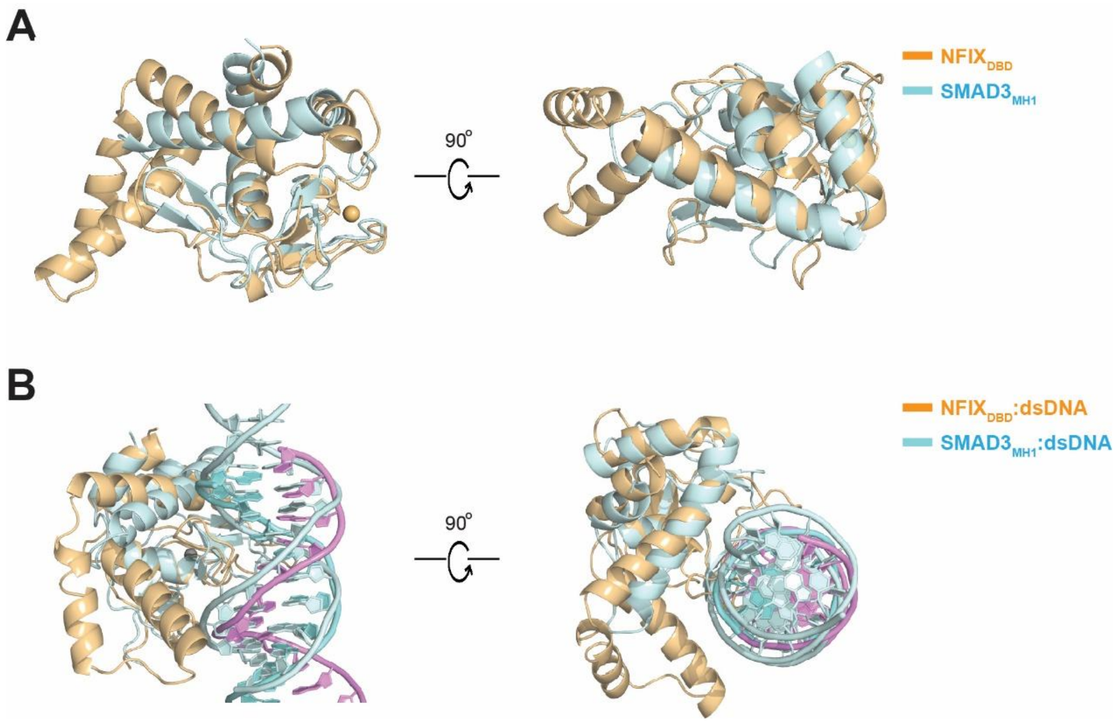

Figure S3. Comparison of NFIX$_{DBD}$ and SMAD$_{MH1}$. A, Superposition of NFIX$_{DBD}$ with the SMAD3 MH1 domain (PDB ID: 1MHD) reveals pronounced divergence, including an extended α-helix region in NFIX. **B**, Comparison of NFIX$_{DBD}$:dsDNA and SMAD3$_{MH1}$:dsDNA complexes reveals distinct modes of DNA recognition. SMAD3 MH1 inserts a β-hairpin into the major groove, whereas NFIX engages the groove predominantly through a DNA-binding loop.

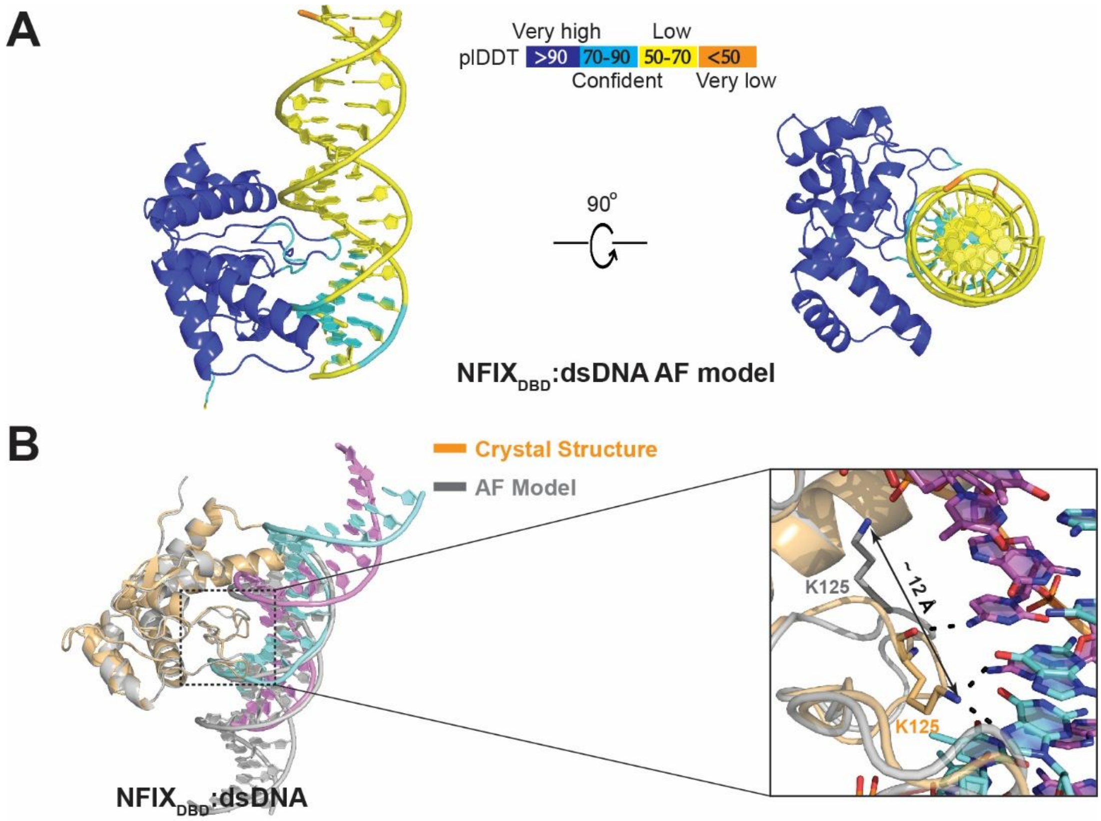

Figure S4. AlphaFold3 fails to recapitulate the NFIX:dsDNA complex structure. **A**, AlphaFold3-predicted model of the NFIX$_{DBD}$:dsDNA complex, displaying low confidence in the DNA-bound region. **B,** Superposition with the crystal structure shows mispositioned DNA and loss of key base-specific contacts required for recognition.

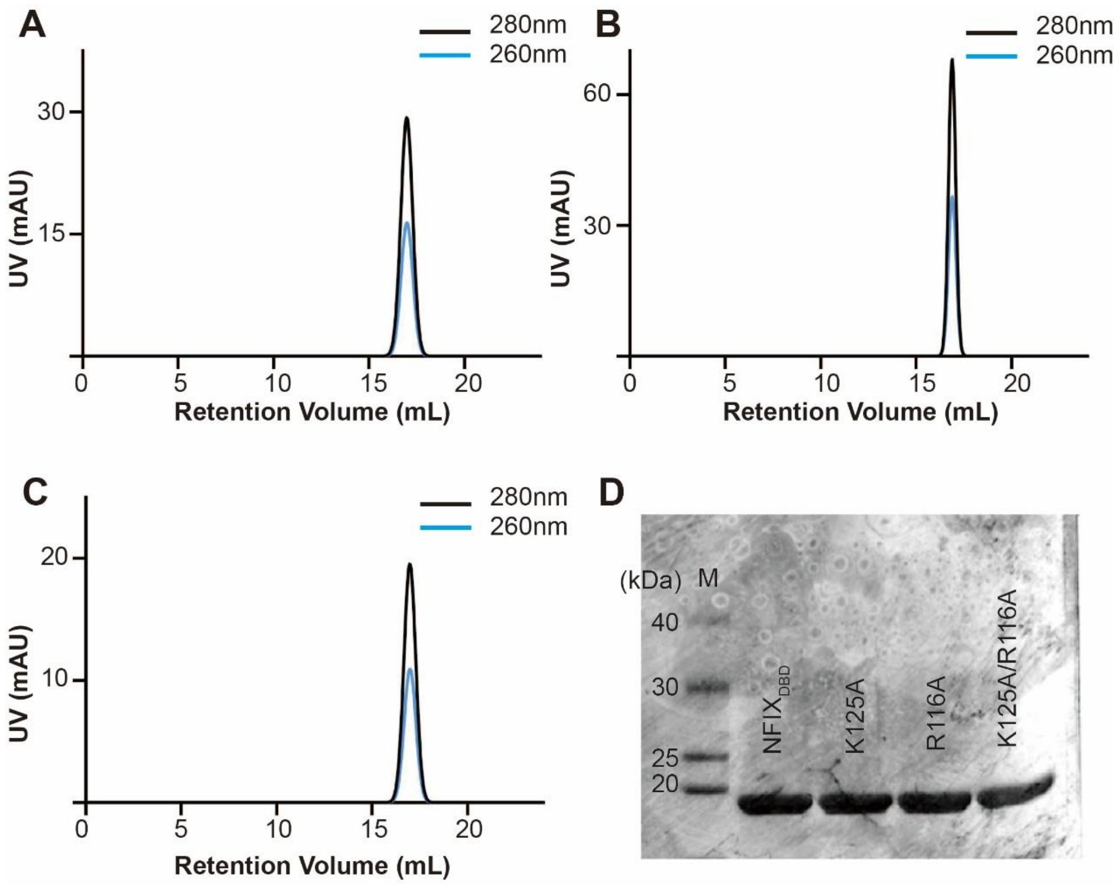

Figure S5. Biophysical quality control of NFIX$_{DBD}$ mutants. A-C, Size-exclusion chromatography (SEC) profiles of R116A (**A**), K125A (**B**), and R116A/K125A (**C**). **D**, Coomassie-stained SDS–PAGE of peak fractions.

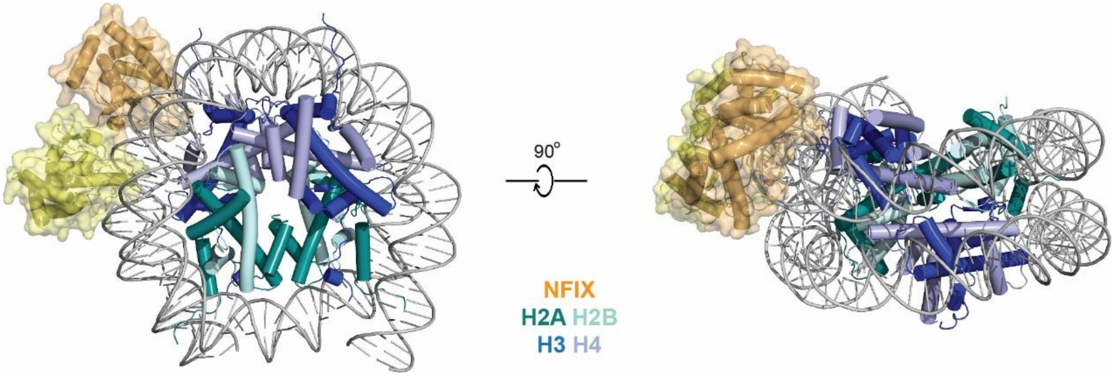

Figure S6. Chromatin-enabled model of potential NFIX dimer engagement. Rigid-body placement of two NFIX DBD molecules (yellow and orange) onto a palindromic site embedded in nucleosomal DNA illustrates how bent, histone-wrapped DNA could accommodate two NFIX molecules without steric conflict. Histones are shown as helices. Two orthogonal views highlight the clearance between NFIX and the histone octamer and the widened major-groove geometry created by DNA curvature. This model is illustrative and supports a context-dependent possibility of chromatin-assisted dimer binding, in addition to the monomeric mode observed on linear B-DNA under our solution conditions.

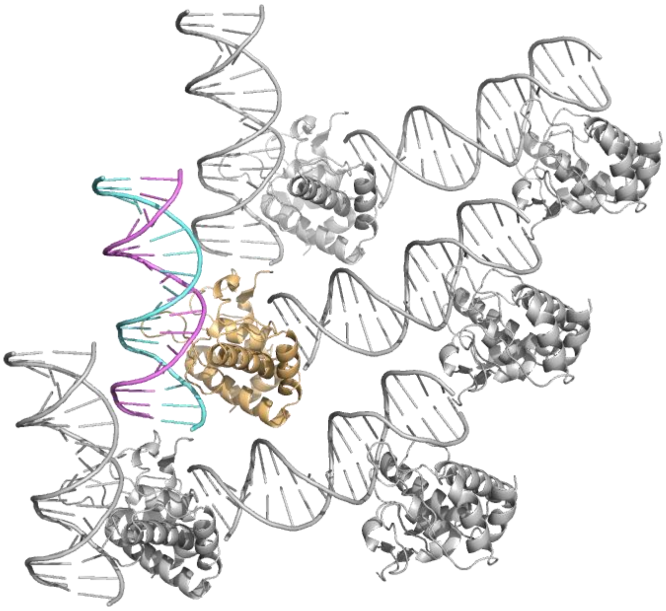

Figure S7. Crystal lattice of the NFIX:dsDNA complex. Packing diagram showing the asymmetric unit $NFIX_{DBD}$:DNA complex in color and symmetry-related neighbors in gray. No base-specific contacts are formed between the ASU and symmetry mates, and lattice interfaces are small and nonspecific, consistent with a 1:1 protein:DNA stoichiometry within the crystal.

Table S1. Binding kinetic parameters derived from the BLI data.

NFIX	Substrate	K_D (nM)	k_{on} ($M^{-1}s^{-1}$)	k_{off} (s^{-1})	R^2
WT	Substrate-1	9.85±0.13	$1.86\pm0.01\times10^{7}$	$1.84\pm0.02\times10^{-1}$	0.9992
WT	Substrate-2	6.01±0.11	$1.92\pm0.01\times10^{7}$	$1.15\pm0.01\times10^{-1}$	0.9993
**$NFIX_{DBD}$**	**Substrate**	**K_D (nM)**	**k_{on} ($M^{-1}s^{-1}$)**	**k_{off} (s^{-1})**	**R^2**
WT	Substrate-1	10.27±0.10	$1.99\pm0.01\times10^{7}$	$2.05\pm0.02\times10^{-1}$	0.9993
WT	Substrate-2	8.75±0.12	$2.16\pm0.09\times10^{7}$	$1.89\pm0.02\times10^{-1}$	0.9991
R117A	Substrate-2	No binding	NA	NA	NA
K125A	Substrate-2	No binding	NA	NA	NA
R116A/K125A	Substrate-2	No binding	NA	NA	NA

Table S2. Data collection and refinement statistics.

	$NFIX_{DBD}$:dsDNA
**Deposition**	
PDB entry code	9WA7
**Data collection**[a]	
Space group	$P2_12_12$
Cell dimensions	
a, *b*, *c* (Å)	77.0, 93.3, 41.9
α, β, γ (°)	90, 90, 90
Resolution (Å)	40.00-2.30 (2.38-2.30)[b]
R_{pim}	0.059 (0.264)
$CC_{1/2}$	0.942 (0.743)
CC^*	0.985 (0.923)
$I / \sigma I$	12.24 (2.13)
Completeness (%)	99.9 (99.3)
Redundancy	9.9 (8.7)
**Refinement**	
Resolution (Å)	31.18-2.31 (2.43-2.31)
No. reflections	13,756
R_{work} / R_{free}	0.226/0.258
No. atoms	
Protein	2,178
Ligand/ion	733
Water	105
B-factors	
Protein	44.8
Ligand/ion	53.3
Water	47.1
R.m.s. deviations	
Bond lengths (Å)	0.003
Bond angles (°)	0.52
Ramachandran plot	
Favored (%)	98.1
Allowed (%)	1.9
Disallowed (%)	0.0

[a)]One crystal was used for data collection.

[b)]Values in parentheses are for highest-resolution shell.

Table S3. Detailed interactions between $NFIX_{DBD}$ and DNA.

$NFIX_{DBD}$ (residue/chain)	Atom	dsDNA (residue/chain)	Atom	Distance (Å)
Arg38/A	NH1 [N]	Gua12/C	OP2 [O]	3.25
Arg38/A	NH2 [N]	Gua12/C	OP2 [O]	3.19
Lys78/A	NZ [N]	Thy3/B	OP1 [O]	2.83
Lys81/A	NZ [N]	Thy4/B	OP1 [O]	2.89
Gln110/A	NE2 [N]	Gua5/B	OP2 [O]	3.36
Gln110/A	NE2 [N]	Gua5/B	O5′ [O]	3.01
Arg116/A	NH1 [N]	Gua12/C	O6 [O]	3.46
Arg116/A	NH2 [N]	Gua12/C	N7 [N]	2.81
Arg121/A	NH1 [N]	Gua2/B	OP1 [O]	2.57
Ala123/A	O [O]	Cyt14/C	N4 [N]	2.57
Lys125/A	NZ [N]	Gua5/B	N7 [N]	2.85
Lys125/A	NZ [N]	Gua6/B	O6 [O]	3.02
Lys125/A	O [O]	Cyt13/C	N4 [N]	2.90
Thr145/A	O [O]	Thy11/C	OP1 [O]	3.12

Table S4. Primers sequences used in qPCR.

Gene	Forward primer 5’-3’	Reverse primer 5’-3’
NFIX	TACCAGCAGCGTGATGAGTT	CTCCTCGTCCTTCGACATCC
NMNAT2	ACGGTGATGCGGTATGAAGAG	CACCTCCATATCTGCCTCGTT
PPARD	CAGGGCTGACTGCAAACGA	CTGCCACAATGTCTCGATGTC
IL6	ACTCACCTCTTCAGAACGAATTG	CCATCTTTGGAAGGTTCAGGTTG
EGR1	GGTCAGTGGCCTAGTGAGC	GTGCCGCTGAGTAAATGGGA
IL1RN	CATTGAGCCTCATGCTCTGTT	CGCTGTCTGAGCGGATGAA
NDRG2	AGACTCACTCTGTGGAGACAC	CGTGGTAGGTAAGGATCGCTG
